# Impact of pre-crosslinks on the self-transformation performance of thermoplastic polyesters into vitrimers *via* intermolecular transesterification†

**DOI:** 10.1039/d4ra08285k

**Published:** 2025-01-06

**Authors:** Yuhka Shimizu, Mikihiro Hayashi

**Affiliations:** a Department of Life Science and Applied Chemistry, Graduate School of Engineering, Nagoya Institute of Technology Gokiso-cho, Showa-ku Nagoya Aichi Japan 466-8555 evh70675@ict.nitech.ac.jp

## Abstract

We recently proposed a concept of self-transformation from thermoplastic polyesters into vitrimers *via* intermolecular bond exchange as the cross-linking reaction. Key was the use of polyesters bearing hydroxyl side groups, which were cross-linked without additional cross-linkers through intermolecular transesterification in the presence of a suitable catalyst. In our previous study, a linear polyester was synthesized as the starting polymer by reacting dithiol monomers containing ester bonds (2-SH) with diepoxy monomers (2-epoxy) *via* a thiol–epoxy reaction, generating hydroxyl side groups along the polyester chain. In the present study, we introduce tetra-functional epoxy monomers (4-epoxy) into the starting materials, with the proportion of 4-epoxy limited to be below the experimentally determined percolation threshold. Consequently, the initial polyester still behaves as a thermoplastic, while the 4-epoxy units serve as pre-crosslinks. We evaluate the impact of 4-epoxy on bond-exchange-based material transformation, based on the thermal, mechanical, and rheological analyses. The important findings are improvements in the time required for transformation and enhancements in the mechanical properties of the final cross-linked products, which could contribute to the practical application of the bond-exchange based transformable materials.

## Abstract 2

The performance of self-transformation from thermoplastic polyesters into vitrimers *via* intermolecular transesterification is improved by incorporating pre-crosslinks in the initial polyesters.

## Graphical abstract

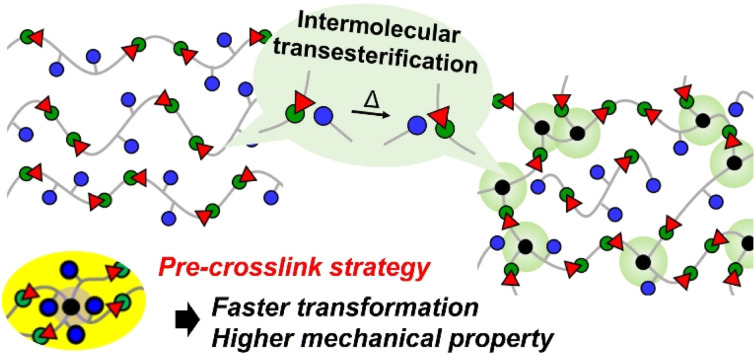

## Introduction

Various concepts have been developed to address the non-sustainability of conventional cross-linked polymers with permanent covalent bonds.^1,2^ The concept of covalent adaptable networks (CANs), which employ dynamic covalent bonds, has emerged as a valuable option,^3,4^ allowing for the connectivity of network strands to be altered in response to suitable external stimuli. This enables the relaxation and diffusion of network strands, facilitating sustainable functions such as recyclability and healability in cross-linked polymers. Vitrimers represent a new class of CANs,^5,6^ characterized by their associative bond exchange mechanism, which preserves network connectivity during the bond exchange process. When subjected to appropriate stimuli, typically heat, vitrimers behave like thermoplastics; however, the viscosity–temperature relationship of vitrimers follows an Arrhenius dependence, resulting in a less pronounced viscosity reduction with increasing temperature compared to typical thermoplastics.^7^ This weak temperature dependence of viscosity reduction endows vitrimers with unique malleability, allowing vitrimer films to be reshaped into complex forms without flow.

**Fig. 1:**
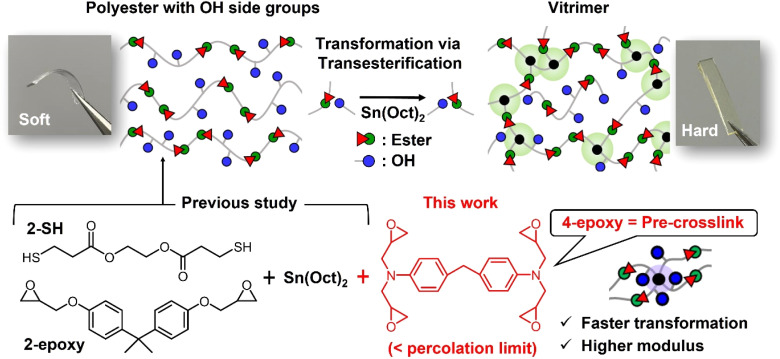
The molecular design for self-transformation of thermoplastic polyesters into vitrimers. Effects of 4-epoxy as the pre-crosslink in the initial polyester on the transformation performance is investigated in this work.

In addition to the conventional benefits of bond exchange associated with covalent adaptable networks (CANs) or vitrimers, recent studies have demonstrated material transformations facilitated by bond exchange.^8–11^ For instance, T. Xie *et al.* designed networks featuring pendant hydroxyl (OH) groups and polyester graft chains both attached to the strands.^12,13^ The associative bond exchange *via* transesterification between the OH groups and grafted polyester chains enabled topological reprogramming, leading to an alteration of mechanical properties in a post-cross-linking manner. We recently reported a novel transformation from thermoplastic to cross-linked polymers utilizing intermolecular transesterification as the cross-linking mechanism (Fig. 1).^14^ Polyesters with abundant OH side groups were synthesized through polymerization of a dithiol monomer containing ester bonds and a diepoxy monomer in the presence of stannous octoate (Sn(Oct)_2_). Notably, Sn(Oct)_2_ served not only as a catalyst for the thiol–epoxy reaction but also as a catalyst for transesterification. The polymerization was conducted at 100 °C, resulting in a linear polyester without cross-linking. Upon heating the obtained polyester to temperatures above 130 °C, cross-linking progressed *via* intermolecular transesterification. Consequently, the initial linear polymers were transformed into cross-linked polymers, allowing material properties to be adjusted simply by varying the degree of bond exchange. We also demonstrated that this self-transformation enabled application as a cross-linkable hot melt adhesive. Importantly, transesterification remained active in the final cross-linked state, allowing the cross-linked materials to behave as vitrimers. This feature is advantageous compared to other conventional self-transformative polymers that form permanent covalently bonded cross-links.^15–18^

However, in our previous system, the transformation rate was still slow; the gelation time (*i.e.*, time for the network formation) at 170 °C, determined by time-resolved rheology, exceeded 1 h significantly. For practical applications, it is essential to shorten the time required for transformation. To address this, we drew insights from existing knowledge on pre-crosslink strategies. In general, cross-linking materials exhibit a percolation point where polymer chains become interconnected, forming a continuous network. At this percolation point, while some cross-linkable groups may remain unreacted, the material starts to display properties typical of cross-linked polymers, such as non-flowability and nonsolubility. Therefore, employing pre-crosslinked polymers just before reaching the percolation limit can significantly shorten the practical cross-linking time, where only small progress of the cross-linking reaction can provide the percolated network polymers. This strategy has been effectively implemented to enhance the practical application of functional gel materials, where the reaction conversion is precisely controlled to obtain the pre-gelation components.^19,20^

In this study, we designed pre-crosslink components using tetra-functional epoxy molecules (Fig. 1). In our previous design, we utilized only dithiol and diepoxy monomers to obtain the starting polyesters. The incorporation of tetra-functional epoxy units introduces pre-crosslink points (or branch points) within the starting polyesters. It is important to note that the fraction of 4-epoxy is limited to be below experimentally determined percolation threshold, preserving the thermoplastic nature, specifically the flowability of the material. In this context, the presence of pre-crosslink points can not only shorten the practical gelation time but also enhance mechanical properties, as the 4-epoxy units can function as additional cross-links in the final network. We evaluate the transformation performance from the initial thermoplastic polyesters to vitrimers, focusing on the actual cross-linking time as well as thermal and mechanical properties. Overall, this study provides valuable insights for the development of practical transformable polymers utilizing bond exchange-triggered cross-linking.

## Experimental section

### Materials

Ethylene glycol bis(3-mercaptopropionate) (2-SH), 2,2-bis(4-glycidyloxyphenyl)propane (2-epoxy), 4,4′-methylenebis(*N*,*N*-diglycidylaniline) (4-epoxy), and stannous octoate (Sn(Oct)_2_) were purchased from TCI. These chemicals were used as received.

### Polymerization and cross-linking

2-Epoxy, 4-epoxy, and Sn(Oct)_2_ were first mixed by stirring in a glass bin at room temperature (*i.e.*, 25 °C) for 10 min. 2-SH was then added, and the mixture was further stirred for 30 min. The molar ratio of 2-SH : 2-epoxy : 4-epoxy was varied to obtain the polyester with a different fraction of 4-epoxy unit (the exact mole ratio is explained in the Result and discussion section). In any case, the mole ratio of SH and epoxy groups were set to be unity, and the mole ratio of Sn(Oct)_2_ to the SH groups was fixed to be 5 mol%. The mixture was poured into a Teflon-made mold, followed by heating at 100 °C for 12 h in an oven. No purification step was made, and thus the obtained polymers still contained Sn(Oct)_2_ catalyst. For the subsequent transformation *via* intermolecular transesterification, the polymer was simply heated at various high temperatures (*i.e.*, 130 °C, 150 °C, and 170 °C) in an oven.

### Gel fraction

The gel fraction (*f*_gel_) was measured as follows; cross-linked samples (*ca.* 0.1 g) were immersed in tetrahydrofuran (THF). The solution was replaced with a new solvent, three times with an interval of 24 h. The final swollen sample was dried by vacuum, and the mass of the dried sample (*m*_d_) was compared with mass of initial mass (*m*_i_). *f*_gel_ was then estimated by the relationship, *f*_gel_ = *m*_d_/*m*_i_ × 100.

### FT-IR spectroscopy

The progress of reaction between the SH and epoxy groups was confirmed by Fourier transform infrared spectroscopy (FT-IR). The measurement was carried out at 25 °C using an FT-IR 4700 spectrometer (JASCO Co.) combined with an attachment of attenuated total reflectance.

### Thermal property investigation

The glass transition temperatures (*T*_g_) were investigated by differential scanning calorimetry (DSC), using a DSC7020 (Hitachi High-Tech). The 1st heating was from 30 °C to 100 °C, 1st cooling was from 100 °C to −50 °C, and the 2nd heating was from −50 °C to 100 °C, where the temperature ramp rate was set to be 10 °C min^−1^. The thermal decomposition temperature (*T*_d_) was investigated by thermogravimetric analysis (TGA), using a STA200 (Hitachi High-Tech). The temperature was increased from 30 °C to 550 °C with a ramp rate of 10 °C min^−1^. DSC and TGA were performed under N_2_ gas flow.

### Temperature-sweep rheology

For the polyesters before cross-linking, the measurement was performed, using a shear-type rheometer, MCR102e (Anton Paar), and disposable parallel plates with a diameter of 8 mm. In any case, the frequency and strain were set to be 1 Hz and 0.1%, respectively. The temperature was increased from −30 °C to 160 °C with a ramp rate of 5 °C min^−1^. For the cross-linked samples, the measurement was performed, using a uniaxial rheometer DMA850 (TA Instruments). Rectangular-shaped samples with thickness of 1 mm, width of 4 mm, and length of 20 mm were prepared using a razor blade. The frequency and strain were fixed to be 1 Hz and 0.1%, respectively. The temperature was increased from −30 °C to 200 °C with a ramp rate of 10 °C min^−1^. The measurements of shear and uniaxial rheometers were all conducted under N_2_ gas flow.

### Isothermal rheology

The progress of network formation was assessed by performing a time-resolved isothermal rheology test, using a shear-type rheometer, MCR102e (Anton Paar), and disposable parallel plates with a diameter of 8 mm. The measurement was conducted with a fixed frequency of 1 Hz and strain of 3% under constant temperature conditions, *i.e.*, 130 °C, 150 °C, and 170 °C. The measurements were all conducted under N_2_ gas flow.

### Tensile properties

The tensile properties were investigated using an AGS-500NX (SHIMADZU). Dumbbell-shaped samples with thickness of 1 mm, gauge length of 16 mm, and gauge width of 4 mm were prepared using a cutting die. The test was carried out at an elongation rate of 10 mm min^−1^ at 25 °C.

### Vitrimer properties

The stress relaxation tests were conducted with MCR102e (Anton Paar), using disposable parallel plates with a diameter of 8 mm. The strain was 5%, and the temperature was 180 °C. The temperature-ramp creep tests were performed with a TMA7100 (Hitachi High-Tech). The temperature was increased from 100 °C to 200 °C at a ramp rate of 5 °C min^−1^, where a constant small force of 10 kPa was applied. Both measurements were carried out under N_2_ gas flow.

## Results and discussion

As shown in Fig. 1, the starting polyesters were synthesized *via* thiol–epoxy reactions^21,22^ (see details in the Experimental section). In our previous study, a linear polyester with multiple OH side groups was synthesized using only a dithiol monomer bearing ester bonds (*i.e.*, 2-SH) and a diepoxy monomer (*i.e.*, 2-epoxy), where the OH side groups were generated from the thiol–epoxy reaction. In this study, we introduced a tetra-functional epoxy monomer (*i.e.*, 4-epoxy), which works as pre-crosslinks and thus provides branched polyesters with multiple OH side groups. The polymerization was conducted in the presence of Sn(Oct)_2_, which also served as a transesterification catalyst in the later cross-linking reaction. The feed ratio of monomers was set to 2-SH : 2-epoxy : 4-epoxy = 100 : 100 − 2*X*: *X*, where *X* ranged from 0 to 20; thus, the ratio of epoxy to thiol was maintained at unity. The factor of 2 in 2*X* accounts for the two-fold epoxy units present in 4-epoxy compared to 2-epoxy. For example, when *X* = 10, the ratio of thiol to epoxy becomes 100 × 2 : (80 × 2 + 10 × 4) = 1 : 1. In all cases, the fraction of Sn(Oct)_2_ was maintained at 0.05 equivalent to the thiol groups. The bulk mixture was heated at 100 °C for 12 h, yielding four types of starting polymers with varying fractions of 4-epoxy. The polyesters are designated as P-*X*, where *X* represents the *X* in the above feed ratio of 2-SH : 2-epoxy : 4-epoxy = 100 : 100 − 2*X* : *X* (see the actual feed mole ratio and feed weight ratio in ESI, Table S1†).

**Fig. 2:**
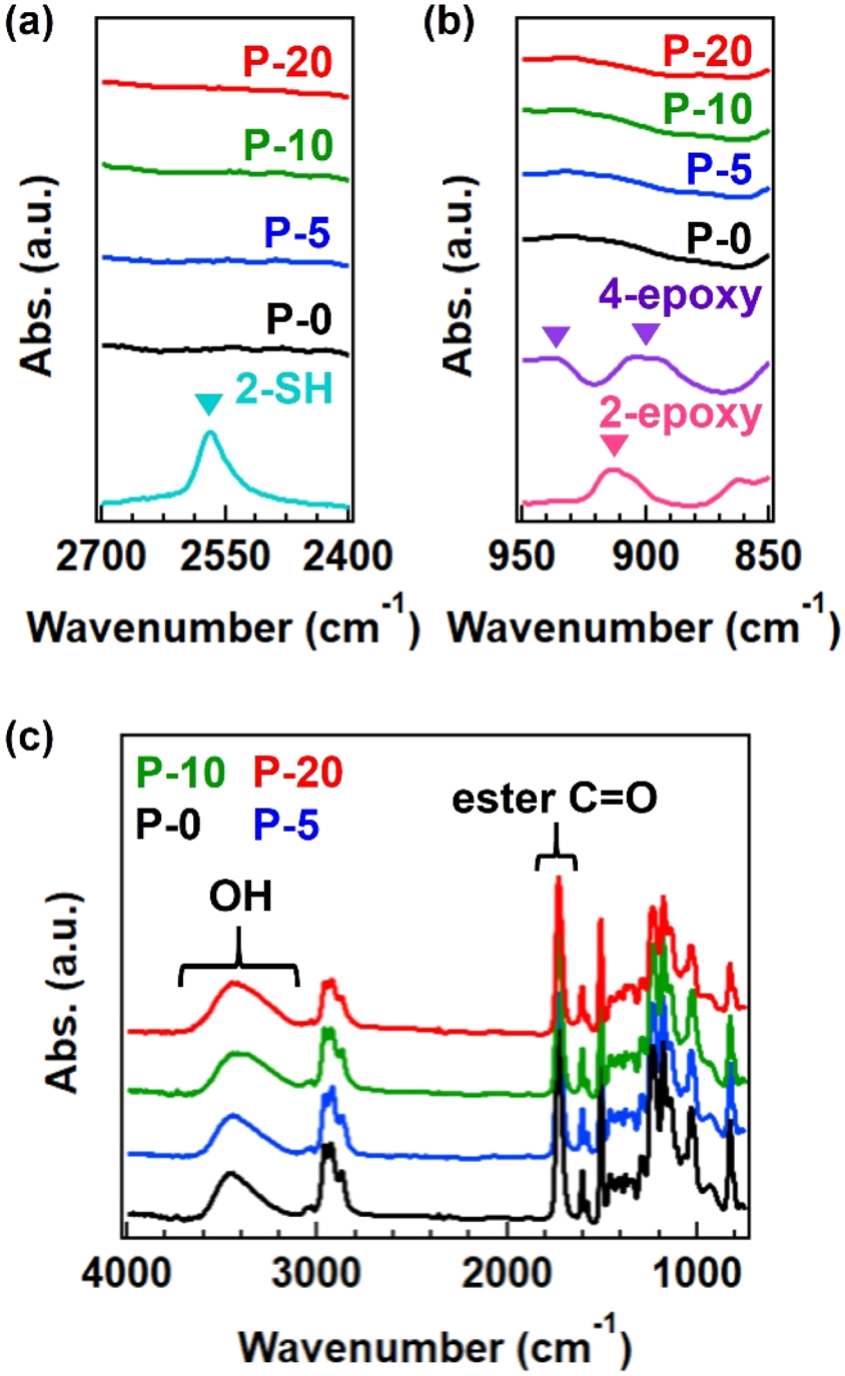
FT-IR spectra for polyesters with various fractions of 4-epoxy at (a) the SH signal region, (b) the epoxy signal region, and (c) the whole region. In (a) and (b), the spectra of starting monomers (2-SH, 2-epoxy, and 4-epoxy) are also provided for comparison. The inverse triangle represents the characteristic signals from SH and epoxy groups. In (c), the spectra are normalized by C=O signal of ester bonds.

The progress of the reaction between thiol and epoxy was confirmed by FT-IR analysis. Although there is a detection limit, the signals from thiol and epoxy groups disappeared after the reaction (Fig. 2a and b), and a new peak corresponding to OH groups was observed (Fig. 2c).^14^ The split signals of epoxy in the spectrum of 4-epoxy was usual, according to the literature,^23^ which has the origin of antisymmetric stretch of epoxy ring. The obtained polymers were soluble in organic solvents such as tetrahydrofuran and *N*,*N*-dimethylformamide; however, a small gel fraction was observed in the case of P-20 (see the summary of gel fraction in Table S2†). The completely dissolved samples, P-0, P-5, and P-10, were analyzed by SEC (Fig. S1 and Table S4†) and ^1^H-NMR (Fig. S2†), confirming the generation of polymers with a number-average molecular weight (*M*_n_) of 7k or 8 k, independent of the fraction of 4-epoxy. Notably, obvious variation was found in the weight-average molecular weight (*M*_w_), increasing from 11k to 22k with an increase in 4-epoxy. Consequently, the dispersity index was gradually enlarged. These facts imply the generation of branched polymers when there are 4-epoxy monomers in the polymerization.

**Fig. 3:**
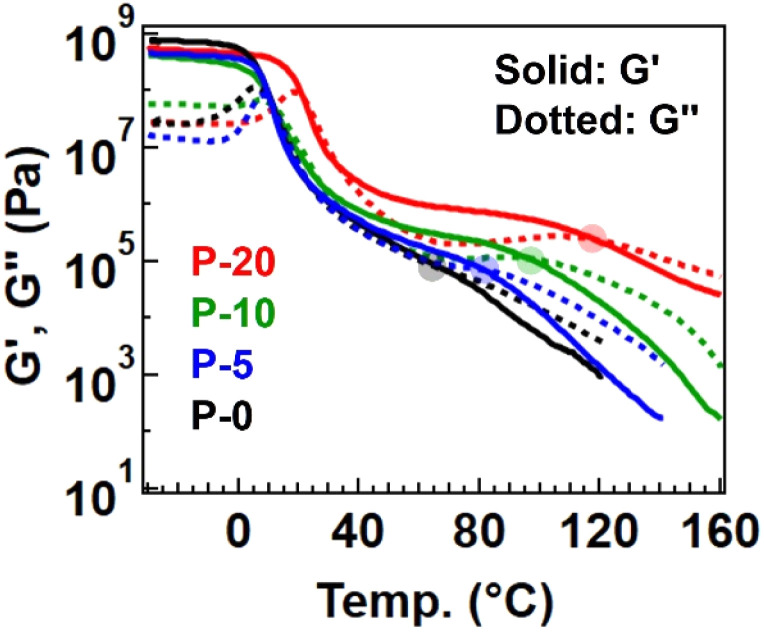
Temperature-sweep rheology data for the polyesters with different fractions of 4-epoxy.

Therefore, the physical properties differed among the samples. First, Fig. 3 summarizes the temperature-sweep rheology results for comparison (with individual data presented in Fig. S3† for clarity). The rubbery plateau gradually became obvious with increasing the fraction of 4-epoxy, accompanied by a rise in the plateau modulus. Additionally, the flow temperature, defined as the intersection of the storage modulus (*G*′) and loss modulus (*G*′′), was shifted to a higher temperature for samples with a greater fraction of 4-epoxy (see the flow temperature in Table S6†). These observed changes indicate that 4-epoxy acted as a connection point between the chains, as intended. All samples exhibited a flow region with *G*′′ > *G*′ at elevated temperatures, despite P-20 containing some gel fraction. Notably, we prepared P-30 to experimentally determine the percolation threshold; for P-30, distinct flow was not observed in the rheological measurements, and the data would rather suggest the properties of a percolated network (Fig. S4 and S5†). The gel fraction after polymerization of P-30 was notably high, 76%. Therefore, we concluded that P-20 contained the maximum fraction of 4-epoxy before establishing a percolated network.

According to classical gelation theory, only a partial presence of 4-epoxy in the starting monomers could potentially generate a percolated network, assuming complete reaction conversion.^24,25^ In contrast, the present series of samples, even P-20, displayed flowability. For the interpretation, we first checked the presence of impurities in the starting monomers, whereas the purity was sufficient (Fig. S6–S8†). Thus, the incomplete network formation in the thiol–epoxy ring opening polymerization with 4-epoxy should imply the incomplete reaction of thiol and epoxy; the progress of the reaction was verified by FT-IR within the detection limit, and the signals from unreacted epoxy or SH groups in ^1^H-NMR were not significantly observed or these were hidden by the signals from polymerized units. More important experimental fact is that the molecular weights of P-0, P-5, and P-10 were not significantly high (*M*_n_ < 10k). This indicates that the actual reaction conversion was not complete, possibly due to the difficulty of the reaction in the high viscosity reaction mixture with bulk monomers. As the polymerization progressed, the viscosity further increased, which provided more difficulty in the completion of reaction, especially at the chain end. Nevertheless, confirming flowability is crucial, as our initial aim is to transform thermoplastic polymers into vitrimers, which necessitates maintaining an easy processable initial state. It is important to note that the larger plateau modulus and higher flow temperature observed in samples with a greater fraction of 4-epoxy indicate an increased fraction of partial pre-crosslink points, which is expected to enhance the cross-linking rate (*i.e.*, transformation rate) for achieving true percolation.

**Fig. 4:**
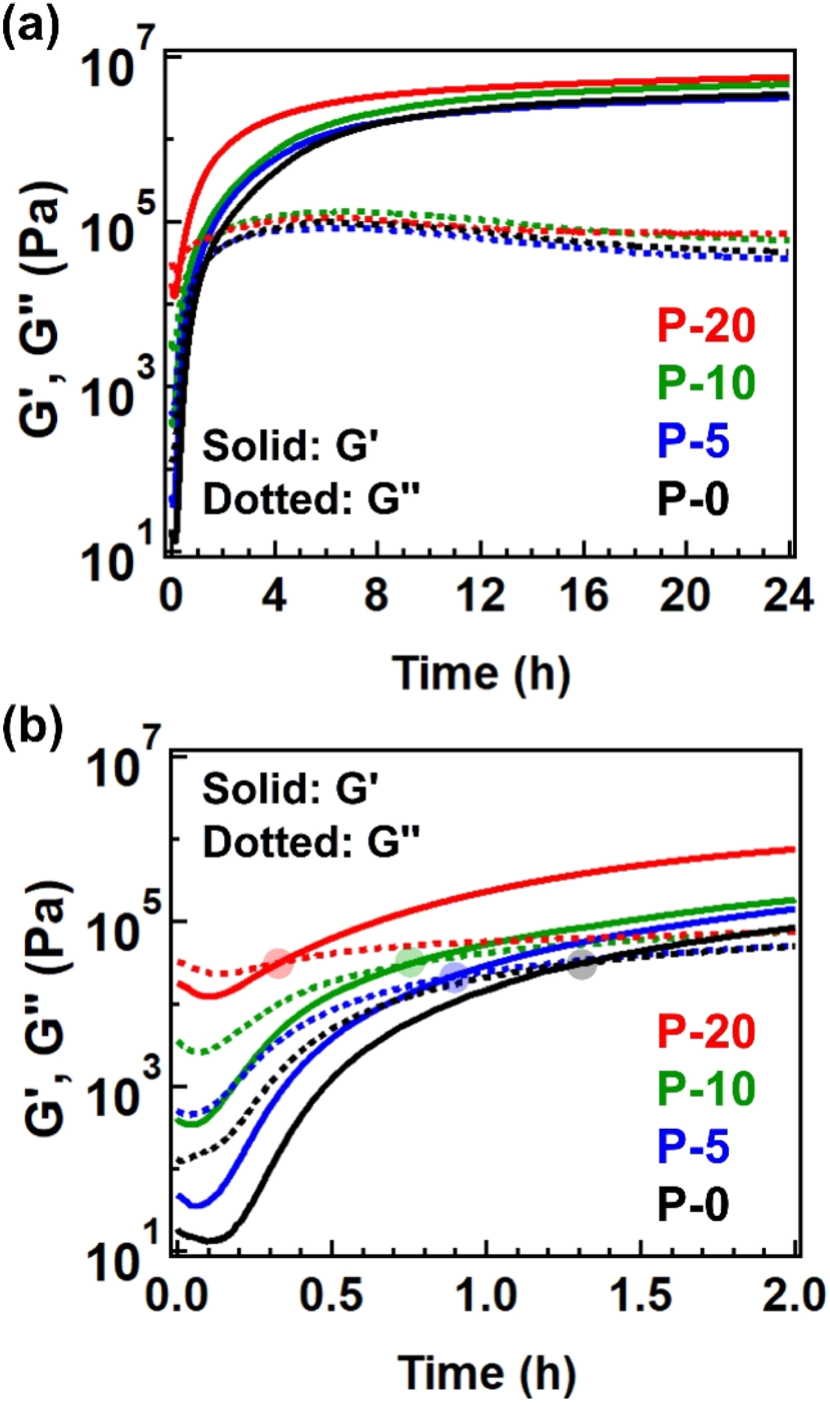
(a) Isothermal time-resolved rheology data measured at 170 °C for 24 h. (b) Expanded data within initial 2 h. The translucent circle represents the cross-point of *G*′ and *G*′′.

We then evaluated this point by conducting time-resolved rheology at 170 °C. The starting polyesters contained Sn(Oct)_2_ as a catalyst for transesterification, allowing the subsequent cross-linking to proceed. Fig. 4a illustrates the changes in *G*′ and *G*′′ during isothermal heating at 170 °C, with expanded data for the initial 2 h provided in Fig. 4b for a clearer observation of the crossover point of *G*′ and *G*′′, defined as the gelation time (*t*_gel_). The *t*_gel_ was observed to be shorter for the sample with a higher fraction of 4-epoxy, specifically 79 minutes for P-0, 53 min for P-5, 45 min for P-10, and 20 min for P-20. Thus, the net cross-linking rate for P-20 was nearly four times greater than that for P-0, attributed to the presence of pre-crosslinks. The same tendency was admitted for the measurements at different temperatures (Fig. S9, S10, and Tables S10–S12†). The shorter *t*_gel_ by increasing the 4-epoxy fraction can be explained as follows. The 4-epoxy unit in the polyesters worked as the pre-crosslink points (or branched point). Thus, before the thermal treatment for full cross-linking, some fractions of chains are already connected. The full cross-linking relies on the intermolecular bond exchange *via trans*-esterification. When there are pre-crosslink points (*i.e.*, the connection point between the chains), the percolation limit is easily achieved; thus, the number of intermolecular bond exchange during the thermal treatment can be reduced for reaching the percolation network, which attains shorted *t*_gel_. The *G*′ value after heating at 170 °C for 24 h was higher for samples with a greater fraction of 4-epoxy, indicating that the 4-epoxy units effectively acted as additional connection points to enhance cross-link density. Notably, all samples exhibited gel fractions of nearly 100% after 24 h of heating at 170 °C (Table S3†). As the gel fraction increased due to the advancement of cross-linking, the glass transition temperature (*T*_g_) also naturally increased (Fig. S11, S12, and Table S13†).

**Fig. 5:**
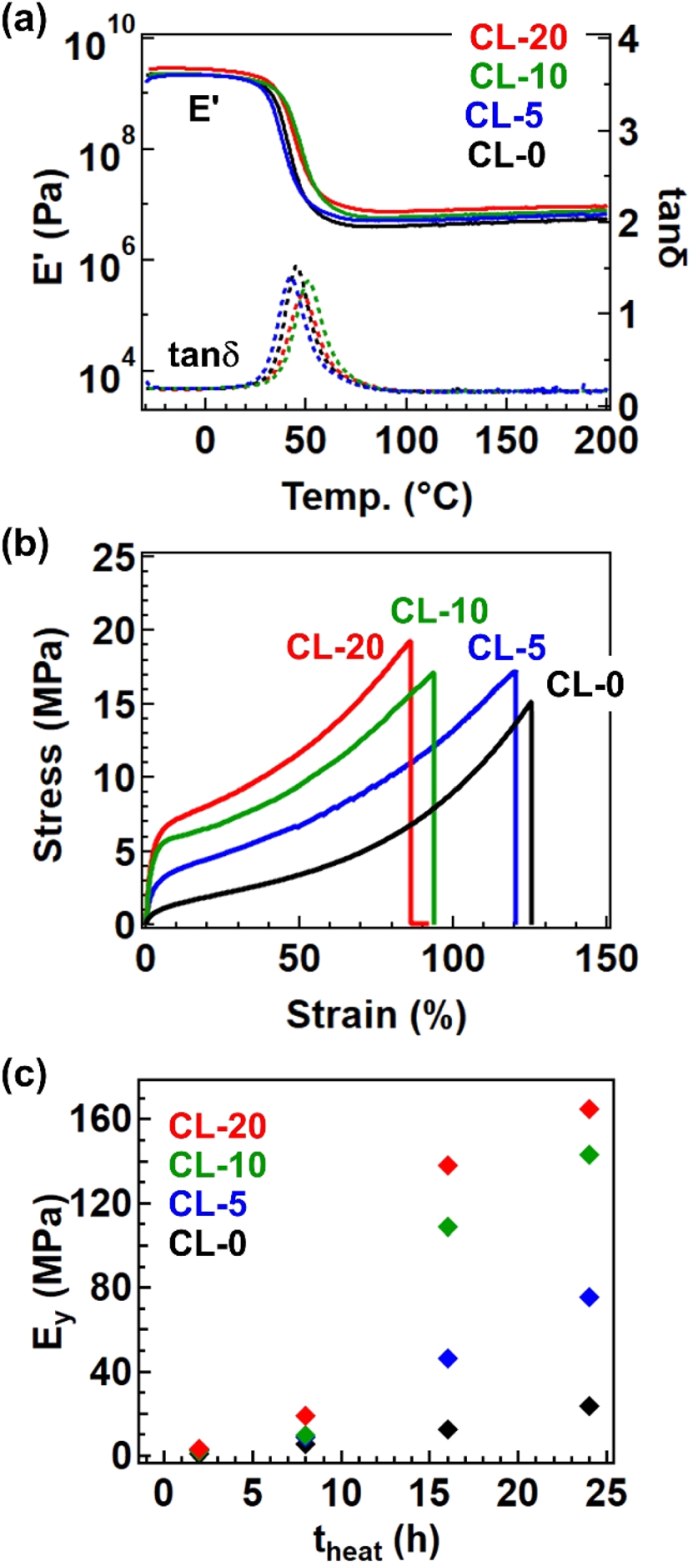
Physical properties of the cross-linked samples after heating at 170 °C for 24 h. (a) Temperature-sweep rheology data and (b) tensile properties. (c) Variation of *E*_y_ as a function of heating time (*t*_heat_).

The formation of the cross-linked structure was further confirmed by temperature-ramp rheology (Fig. 5a). The data presented are for samples after 24 h of heating at 170 °C, where all samples exhibited a stable rubbery plateau up to 200 °C. The cross-linked samples processed by this condition (*i.e.*, 170 °C for 24 h) are hereafter coded CL-*X*, where *X* represents *X* in the precursor polymer P-*X*. The plateau modulus was larger for samples with a greater fraction of 4-epoxy. Tensile tests revealed a significant impact of the 4-epoxy units, particularly on Young's modulus (*E*_y_). Fig. 5b depicts the stress–strain curves for the cross-linked samples, and the variation of *E*_y_ at different heating time (*t*_heat_) was summarized in Fig. 5c. The *E*_y_ was significantly higher for samples with a larger fraction of 4-epoxy; in case of *t*_heat_ = 24 h, CL-20 exhibited a *E*_y_ of 165 MPa, approximately seven times greater than that of CL-0 (*E*_y_. = 23.5 MPa). The substantial difference in *E*_y_ can largely be attributed to the approach of *T*_g_ to the measurement temperature (*i.e.*, room temperature ∼25 °C); in general, the drastic increase in the modulus can be observed at the glass transition regime, by which the small *T*_g_ difference of *ca.* 5 °C provides the significant difference in *E*_y_ for CL-0 and CL-20. Overall, these results clearly indicate that the presence of pre-crosslinks is an effective strategy for enhancing material performance.

**Fig. 6:**
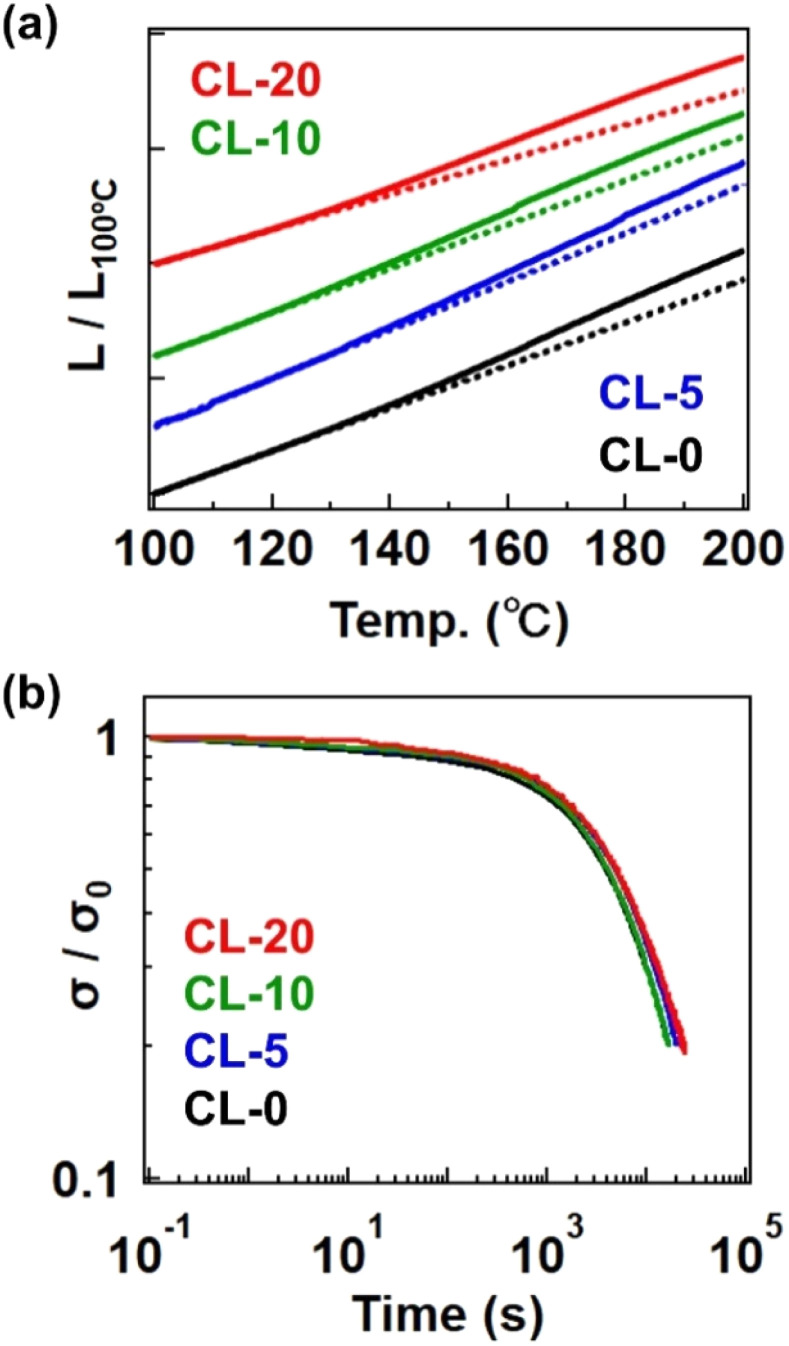
(a) Temperature-ramp creep data. The *Y*-axis represents the sample length (*L*) normalized by the length at 100 °C (*L*_100°C_). The dotted line indicates the approximated straight line extrapolated from low temperature. (b) Stress-relaxation spectra at 180 °C. The *Y*-axis represents the stress (*σ*) normalized by the initial stress (*σ*_0_).

We ultimately confirmed the vitrimer nature by performing stress relaxation and temperature-ramp creep tests for the cross-linked samples, which are standard measurement techniques for assessing the progress of bond exchange within the network.^26,27^Fig. 6a shows data from the temperature-ramp creep experiment, where changes in sample length under a small constant stress (10 kPa) were monitored during heating. The samples exhibited an inflection in length change at *ca.* 130 °C, indicating a softening phenomenon. Given that the *T*_g_ is significantly lower than 100 °C and the decomposition temperature (*T*_d_) is much higher than 300 °C (as shown in the TGA data in Fig. S13†), the observed softening cannot be attributed to either the glass transition or decomposition. Instead, this softening can be interpreted as the activation of bond exchange, a phenomenon typically reported in vitrimer studies. The activation of bond exchange is also reflected in the progress of stress relaxation. Fig. 6b presents data at 180 °C for the four samples, all of which exhibit stress relaxation, further confirming that the cross-linked samples are indeed vitrimers. Initially, we expected the presence of the 4-epoxy unit to adversely affect the relaxation rate due to restricted segment mobility resulting from increased cross-link density.^28,29^ However, the relaxation rates were not significantly different between samples, possibly due to the presence of the tertiary amine unit in 4-epoxy. In our previous study, the tertiary amine in 4-epoxy acted as an internal catalyst,^30,31^ which could compensate for the reduced mobility of strands in samples with a higher cross-link density. While a detailed comparison of bond exchange properties between the samples is beyond the scope of this study, we plan to conduct a comprehensive series of stress relaxation tests in future research focusing on the effects of permanent cross-link fractions on relaxation properties.

## Conclusions

In this study, we revealed some positive effects of incorporating pre-crosslinks into the starting polyesters on the transformation performance of our original self-transformation concept using bond exchange-based cross-linking. The transformation relied on the intermolecular transesterification between ester bonds and OH groups in the starting polyesters. The presence of pre-crosslinks in the starting polyesters shortened the time required for the cross-linking and also enhanced the mechanical properties of the final cross-linked products, as expected. Overall, such transformation systems, especially from thermoplastics into vitrimers, with controlled transformation rate and mechanical properties, should be attractive in the practical sense, which could find the application in the functional adhesives and coatings. Although we focused only on the transformation performance in this study, we will report the details of effects of pre-crosslinks on the vitrimer performance in the near future.

## Data availability

The data supporting this article have been included as part of the ESI.†

## Author contributions

Yuhka Shimizu: data curation, formal analysis, investigation, validation, writing – original draft. Mikihiro Hayashi: formal analysis, methodology, project administration, supervision, validation, visualization, writing – original draft, writing – review & editing.

## Conflicts of interest

There are no conflicts to declare.

## Supplementary Material

RA-015-D4RA08285K-s001
