# Supplementary material for: Impact of pre-crosslinks on the self-transformation performance of thermoplastic polyesters into vitrimers via intermolecular transesterification
Source: RSC Adv. 2025 Jan 6;15(1):524–30. doi: 10.1039/d4ra08285k (PMC11701804; doi:10.1039/d4ra08285k)
Supplement: RA-015-D4RA08285K-s001 [file RA-015-D4RA08285K-s001.pdf]

Supplementary Information (SI) for RSC Advances.
This journal is © The Royal Society of Chemistry 2024

Supporting Information

Impact of pre-crosslinks on the self-transformation performance of thermoplastic polyesters into vitrimers via intermolecular transesterification

Yuhka Shimizu, Mikihiro Hayashi*

Department of Life Science and Applied Chemistry, Graduate School of Engineering, Nagoya Institute of Technology, Gokiso-cho, Showa-ku Nagoya 466-8555, Japan

1. Feed ratio

2. Summary of gel fraction

3. SEC data

4. ^{1}H-NMR of polyesters

5. Temperature-sweep rheology

6. Rheology data for P-30

7. ^{1}H-NMR of starting monomers

8. Isothermal time-resolved rheology data at other temperatures

9. Plot of T_g

10. TGA thermograms

1. Feed ratio

The actual feed mole ratio and feed weight ratio are summarized in Table S1.

Table S1. The feed mole ratio and feed weight ratio

		2-SH	2-epoxy	4-epoxy
P-0	mole ratio	100	100	0
	weight ratio	10.0	14.3	0
P-5	mole ratio	100	90	5
	weight ratio	10.0	12.9	0.9
P-10	mole ratio	100	80	10
	weight ratio	10.0	11.4	1.8
P-20	mole ratio	100	60	20
	weight ratio	10.0	8.6	3.5

2. Summary of gel fraction

Table S2. Summary of gel fraction for polymers

Sample	Gel fraction
P-0	0
P-5	0
P-10	0
P-20	63
P-30	76

Table S3. Summary of gel fraction for cross-linked polymer

Sample	Gel fraction
CL-0	96
CL-5	97
CL-10	98
CL-20	100
CL-30	100

*) The values are for the samples after heating at 170 °C for 24h.

.

3. SEC data

The number average molecular weight (M_n), weight average molecular weight (M_w), and polydispersity index ($Đ$) were determined using size exclusion chromatography (SEC). The set-up was composed of an LC20AD pump system and a RID-20A RI detector (SHIMADZU), where Shodex-gel columns, K-803, K-804, and K-805 (Shodex) were attached. The column temperature was 40 °C, and DMF containing LiBr (0.05 wt%) was used as an eluent at an elution rate of 1.0 mL/min. The standard series of poly(methyl methacrylate) was used for the estimation. SEC data for P-0, P-5, and P-10 are summarized in Figure S1. The values of M_n, M_w and $Đ$ are summarized in Table S4.

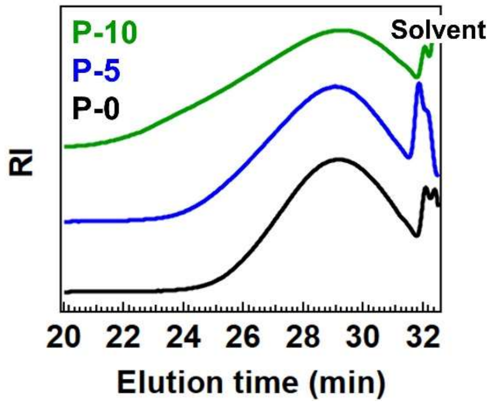

Figure S1. SEC spectra for P-0, P-5, and P-10.

Table S4. Characteristics of polyesters

Sample	M_n	M_w	$Đ$
P-0	6700	11000	1.7
P-5	7900	14000	1.8
P-10	8000	22000	2.7

4. ^{1}H-NMR of polyesters

^{1}H-NMR was measured for the polyesters. The measurement was performed in dimethyl sulfoxide-d_6 with a Bruker Analytik DPX400 spectrometer (400 MHz). Each signal with a different alphabet corresponds to the protons with the same alphabet in the chemical structures shown in the figure.

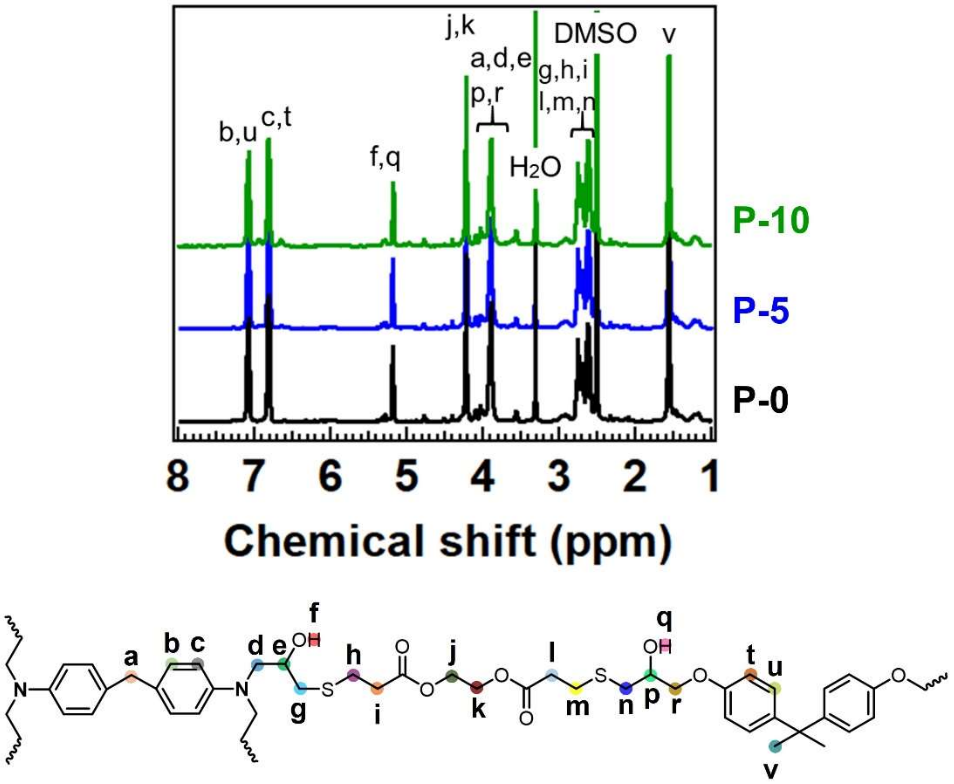

Figure S2. ^{1}H-NMR for polyesters, P-0, P-5, and P-10. The signals in the spectra correspond to the protons with the same alphabet in the chemical structures.

Table S5. Integral ratio of characteristic signals in the ^{1}H-NMR spectra

	a+d+e+p+r	b+u	c+t	f+q	g+h+i+l+m+n	j+k	v
P-0	5.7	4.5	4.5	1.8	11.5	4.0	7.4
P-5	5.4	4.1	4.0	1.6	11.1	4.0	6.9
P-10	5.3	3.8	3.9	1.5	11.4	4.0	6.7

5. Temperature-sweep rheology

Individual data of temperature-sweep rheology for polyesters are provided in Figure S3.

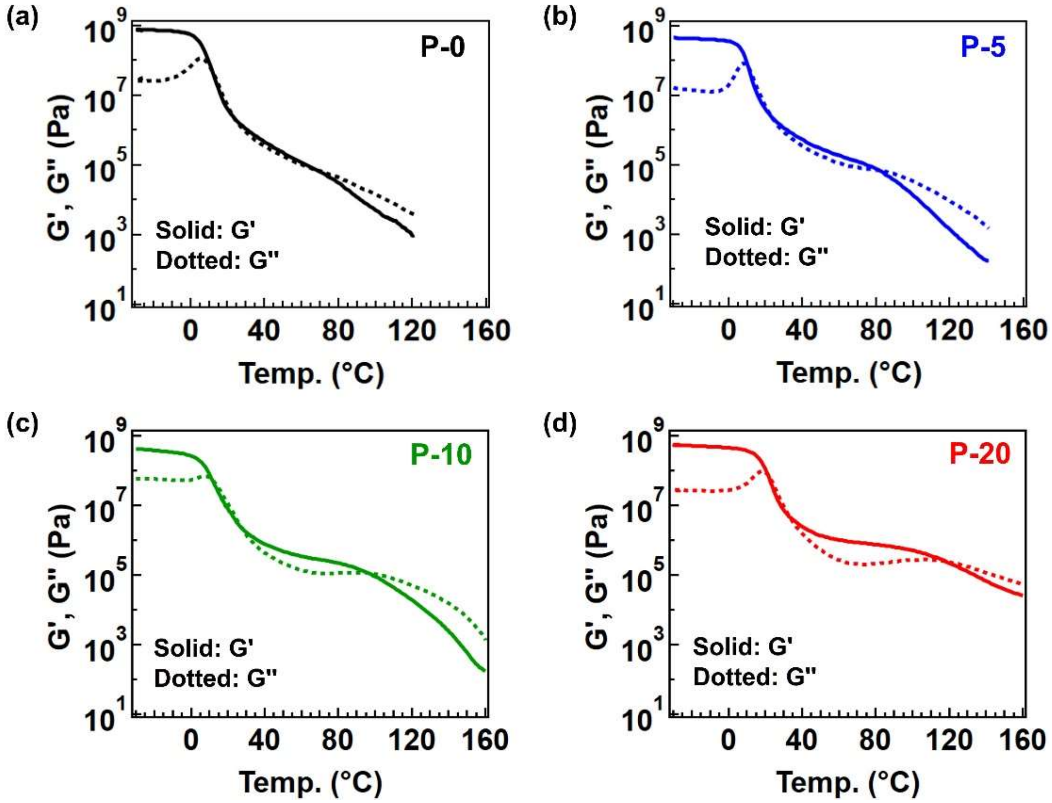

Figure S3. Temperature-sweep rheology data for the polyesters with different fractions of 4-epoxy, i.e., (a) P-0, (b) P-5, (c) P-10, (d) P-20.

Table S6. Summary of flow temperature

	P-0	P-5	P-10	P-20
T_{flow} (ºC)	68	82	97	119

6. Rheology data for P-30

Figure S4 provides a temperature-ramp rheology data for P-30. Unlike other samples with lower fractions of 4-epoxy, P-30 did not exhibit distinct flow region with loss modulus $G'' >$ storage modulus G'. Instead, G' and G'' were always close at high temperature region, which is a characteristic of the polymer near the percolation threshold. Figure S5 represents frequency sweep for P-30 obtained at 80 °C and 90 °C. It has been known that the sample should represent $G' \sim G'' \sim \omega^n$ at the percolation threshold.[S1,S2] Thus, these data indicate that P-30 was very close to the percolated network.

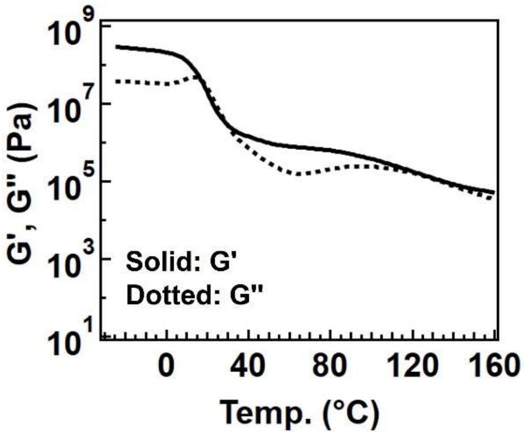

Figure S4. Temperature-sweep rheology data for P-30.

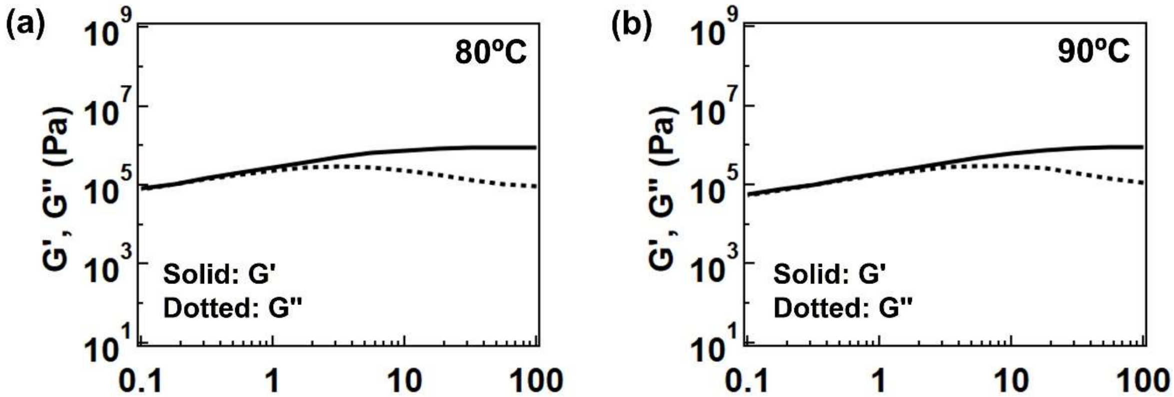

Figure S5. Frequency-sweep rheology data for P-30 at (a) 80 °C and (b) 90 °C.

S1) H. H. Winter, F. Chambon, *J. Rheol.*, 1986, **30**, 367-382.

S2) H. H. Winter, M. Mours, *Adv. Polym. Sci.*, 1997, **134**, 167.

7. ^{1}H-NMR of starting monomers

^{1}H-NMR was measured for the starting monomers, 2-epoxy, 4-epoxy, and 2-SH, to investigate the presence of impurities. The measurement was performed in dimethyl sulfoxide-d_6 with a Bruker Analytik DPX400 spectrometer (400 MHz). Each signal with a different alphabet corresponds to the protons with the same alphabet in the chemical structures shown in the figure.

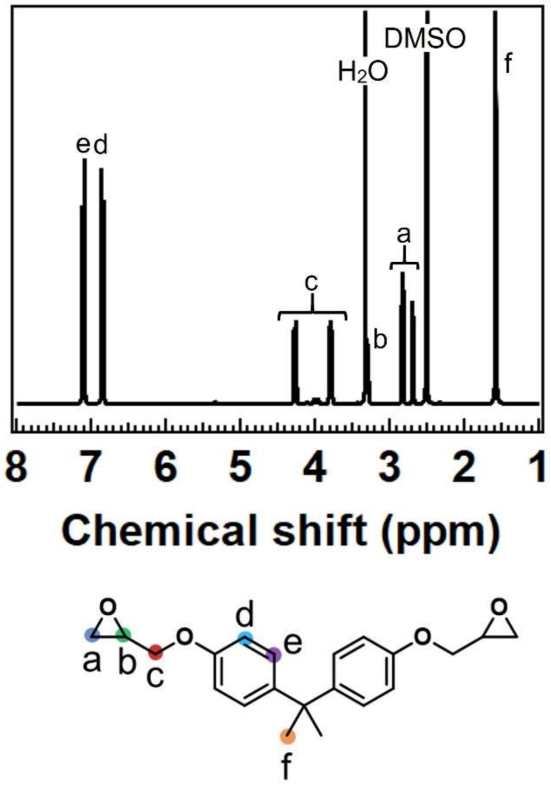

Figure S6. ^{1}H-NMR spectrum for 2-epoxy. The signals in the spectra correspond to the protons with the same alphabet in the chemical structures.

Table S7. Integral ratio of characteristic signals of 2-epoxy

Signal	a	b	c	d	e	f
Integral ratio	3.9	1.9	3.9	4.3	4.3	6.4

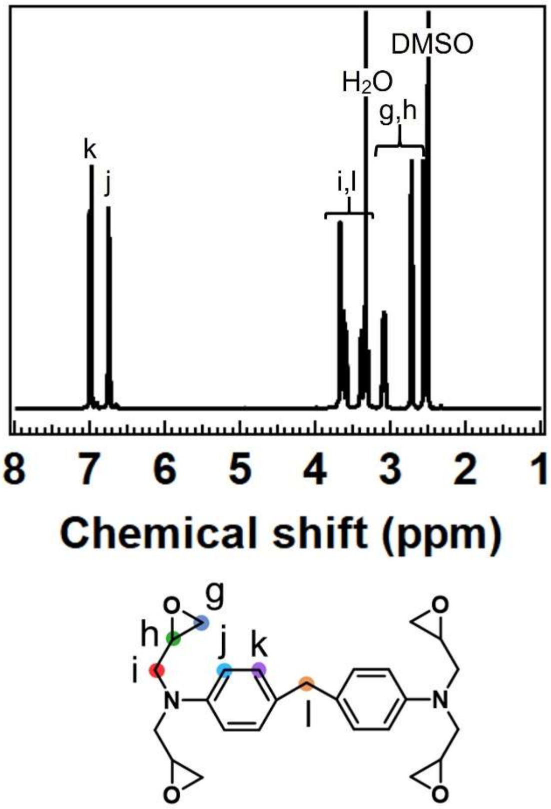

Figure S7. ^{1}H-NMR spectrum for 4-epoxy. The signals in the spectra correspond to the protons with the same alphabet in the chemical structures.

Table S8. Integral ratio of characteristic signals of 4-epoxy

Signal	g+h	i+l	j	k
Integral ratio	11.9	9.9	4.0	4.0

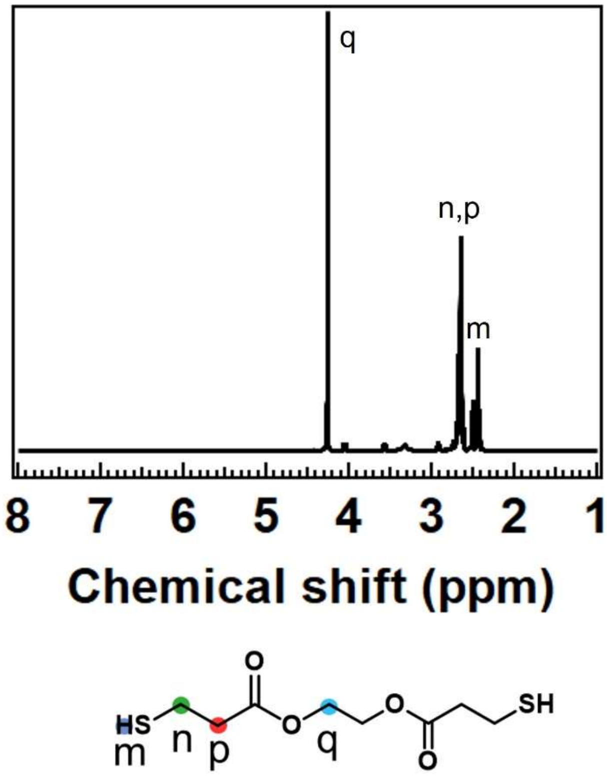

Figure S8. ^{1}H-NMR spectrum for 2-SH. The signals in the spectra correspond to the protons with the same alphabet in the chemical structures.

Table S9. Integral ratio of characteristic signals of 2-SH

Signal	m	n+p	q
Integral ratio	1.7	8.2	4.0

8. Isothermal time-resolved rheology data at other temperatures

Figure S9 represents the isothermal time-resolved rheology data at 150 °C and 130 °C. Figure S10 then summarizes the variation of t_{gel} as function of X in P-X (i.e., the fraction of 4-epoxy) obtained from results at different temperatures.

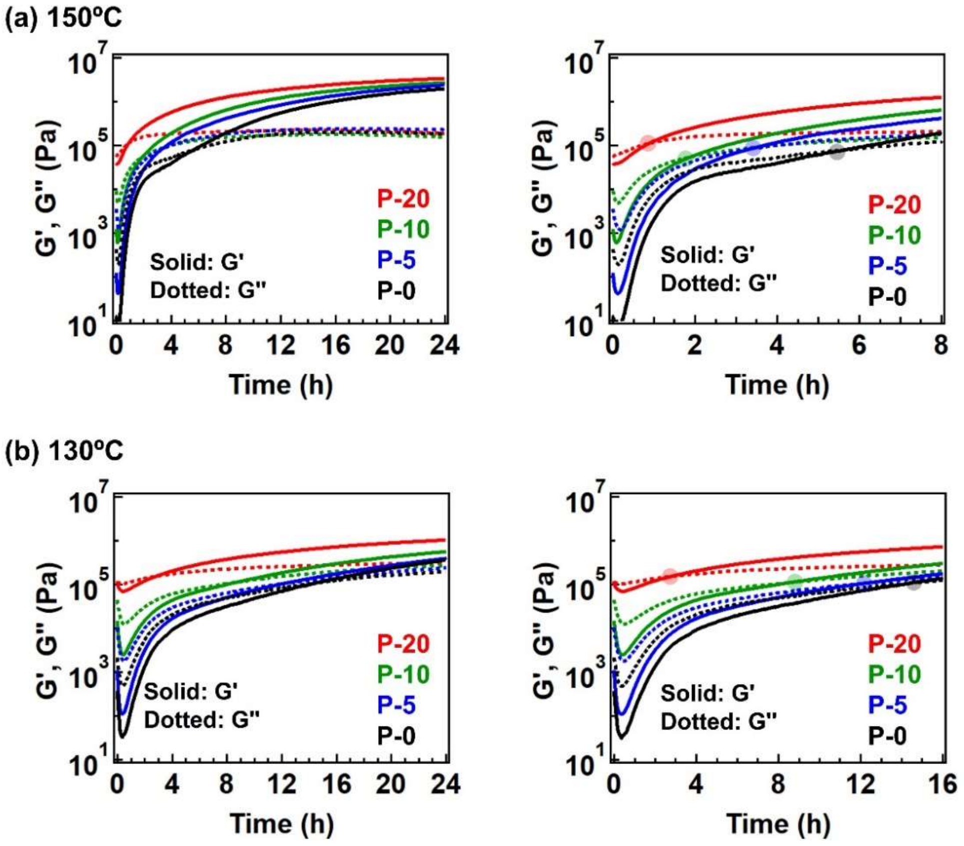

Figure S9. Isothermal time-resolved rheology data measured at (a) 150 °C and (b) 130 °C for 24 h. In the right side, the expanded data are provided for clearer comparison of the cross-point of G' and G''. The translucent circle represents the cross-point of G' and G''.

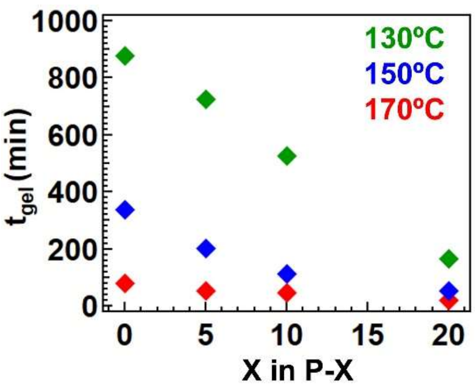

Figure S10. Variation of t_{gel} as function of X in P-X (i.e., the fraction of 4-epoxy) obtained from results at different temperatures.

Table S10. Summary of t_{gel} in heating at 130 °C

	P-0	P-5	P-10	P-20
t_{gel} (min)	876	725	526	167

Table S11. Summary of t_{gel} in heating at 150 °C

	P-0	P-5	P-10	P-20
t_{gel} (min)	337	202	114	53

Table S12. Summary of t_{gel} in heating at 170 °C

	P-0	P-5	P-10	P-20
t_{gel} (min)	79	53	45	20

9. Plot of T_g

Figure S11 summarized the change of T_g as a function of heating time (t_{heat}) at 170 °C. The DSC thermograms of 2nd heating are provided in Figure S12. The Tg values are summarized in Table S13.

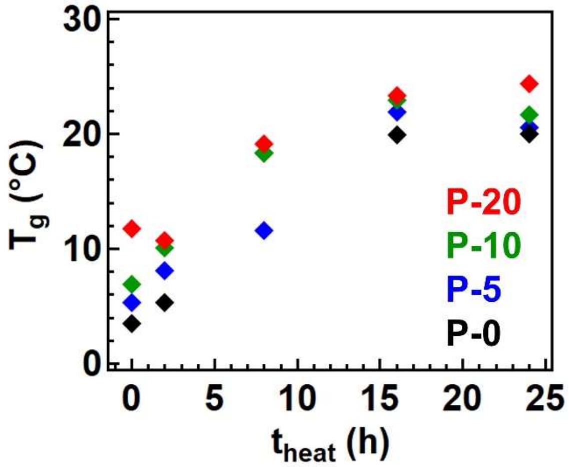

Figure S11. Variation of glass transition temperature (T_g) as a function of heating time (t_{heat}) at 170 °C.

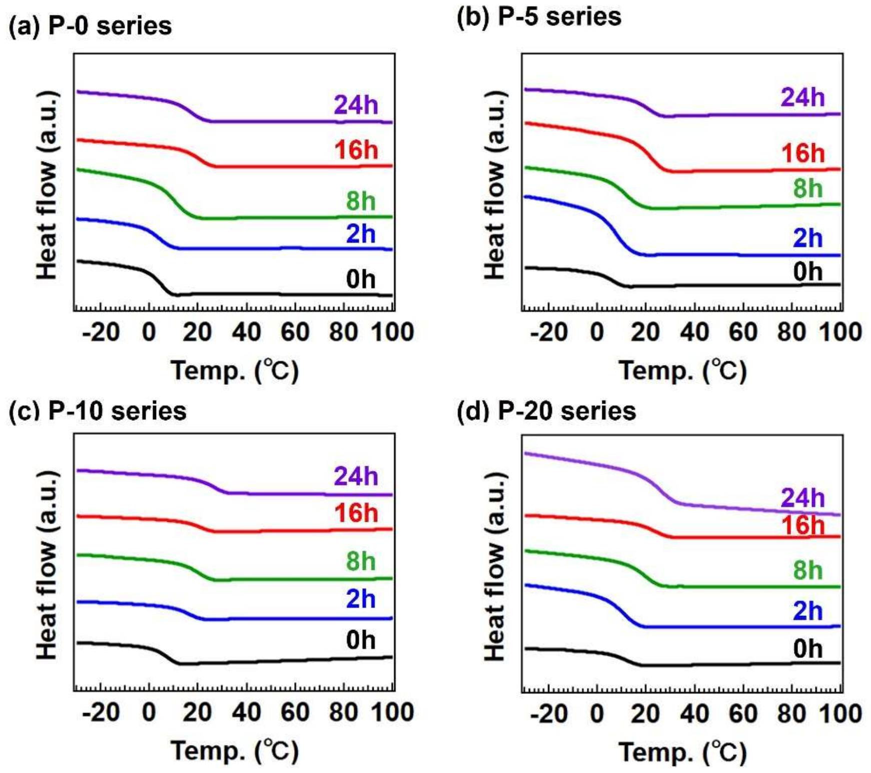

Figure S12. Summary of 2nd heating DSC thermograms for (a) P-0, (b) P-5, (c) P-10, and (d) P-20 series with various t_{heat}.

Table S13. Summary of T_g for the sample after heating at 170 °C for various t_{heat}

t_{heat} (h)	T_g (°C)			
	P-0 series	P-5 series	P-10 series	P-20 series
0	3.5	5.3	6.9	11.7
2	5.3	8.1	10.1	10.7
8	11.2	11.6	18.4	19.1
16	19.9	21.9	22.9	23.3
24	20.0	20.6	21.7	24.4

10. TGA thermograms

Figure S13 represents TGA thermograms for the cross-linked samples, where the cross-linking was performed by heating at 170 °C for 24 h.

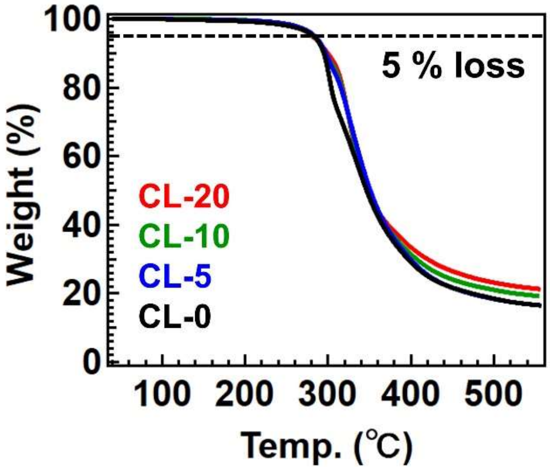

Figure S13. TGA thermograms measured for the sample cross-linked at 170 °C for 24h.
